# Primary breast sarcoma: clinicopathologic series from the Mayo Clinic and review of the literature

**DOI:** 10.1038/sj.bjc.6601920

**Published:** 2004-06-08

**Authors:** C Adem, C Reynolds, J N Ingle, A G Nascimento

**Affiliations:** 1 Department of Anatomic Pathology, Pitie Salpetriere Hospital, Paris, France; 2 Department of Laboratory Medicine and Pathology, Mayo Clinic, Rochester, USA; 3 Division of Medical Oncology, Mayo Clinic, Rochester, USA

**Keywords:** breast, sarcoma, tumour, prognosis, survival, review

## Abstract

Primary sarcomas of the breast are extremely rare, with less than 0.1% of all malignant tumours of the breast. Mayo Clinic Surgical Pathology database was searched for all breast sarcoma from 1910 to 2000. Pathology reports and slides were reviewed and tumour types were determined. Metaplastic carcinomas and phyllodes tumours were excluded. There were 25 women ranging in age 24–81 years (mean 45 years). All but one patient presented with a palpable lump. Mastectomy was performed in 19 patients and lumpectomy in five patients. Histopathological diagnoses were fibrosarcoma (six), angiosarcoma (six), pleomorphic sarcoma (six), leiomyosarcoma (two), myxofibrosarcoma (three), hemangiopericytoma (one) and osteosarcoma (one). Tumour size ranged from 0.3 to 12 cm (mean 5.7). Low-grade lesions were observed in 10 cases and high-grade in 15. Overall, mean follow-up was 10.5 years. Local recurrence was observed in 11 patients and ranged from 2 to 36 months (mean 15 m), while distant metastasis was observed in 10 patients (40%) affecting lungs, bones, liver, spleen, and skin. Of the 25 patients, 12 have died of disease and six of other causes. Five-year overall (OS) and cause-specific survival (CSS) were 66 and 70%, respectively. OS and DFS at 5 years were 91% for tumours ⩽5 cm and 50% for tumours >5 cm. Tumour size was significantly associated with OS (risk ratio=1.3 per 1 cm increase; 95% CI, 1.02–1.7; *P*=0.036). There was no significant difference in OS or CSS between low- and high-grade lesions. In this series, tumour size was a more valuable prognostic factor than tumour grade.

Primary sarcomas of the breast are rare, malignant tumours arising from the mesenchymal tissue of the mammary gland (Oberman, 1965; Barnes and Pietruszka, 1977; Callery *et al*, 1985), with an approximate incidence of 17 new cases per million women (Moore and Kinne, 1996). At the Mayo Clinic, 27 881 malignant breast tumours were seen between 1940 and 1999 (C Adem, personal unpublished data) and 18 breast sarcomas were diagnosed accounting for 0.0006% of breast malignancies.

Breast sarcomas should be distinguished from metaplastic carcinomas (Adem *et al*, 2002). When facing a spindle cell neoplasm in an epithelial organ such as the breast one should be careful in rendering the diagnosis of sarcoma. In this setting, immunohistochemistry using the right antibodies is of major input. Berg *et al* defined stromal sarcomas of the breast in 1962 as a group of mesenchymal malignant tumours with fibrous, myxoid and adipose components, excluding malignant cystosarcoma phyllodes, lymphomas and angiosarcomas (Berg *et al*, 1962). However, series in the literature have included many different entities under the rubric of sarcomas such as cystosarcoma phyllodes, lymphosarcoma and carcinosarcoma (Botham *et al*, 1958; Donegan, 1967; Fawcett, 1967; Kennedy and Biggart, 1967; Rissanen and Holsti, 1968; Gogas *et al*, 1976; Ludgate *et al*, 1977; Khanna *et al*, 1981; Christensen *et al*, 1988; Terrier *et al*, 1989; Pitts *et al*, 1991; Ciatto *et al*, 1992; Luna Vega *et al*, 1992; McGregor *et al*, 1994; Moore and Kinne, 1996; McGowan *et al*, 2000). For this review, we choose to categorise primary breast sarcomas in histogenic terms, similar to other soft-tissue sarcomas, thus including angiosarcomas, and excluding malignant cystosarcomas phyllodes, as reported by others (see Table 1

**Table 1:** Major breast sarcomas comparable series in the English literature

**Author**	***N* cases/period**	**Median age (years)**	**Median size (cm)**	**Diagnosis**	**Prognostic factors**
Barnes and Pietruszka (1977)	10/31 years	51	6.3	5F, 1RMS, 1Le, 2OGS, 1 Li	Tumour contour, atypia, mitosis
Barrow *et al* (1999)	59/43 years	45	UK	32F, 17A, 1OGS, 7 NOS	Size, margins status, type
Berg *et al* (1962)	25/UK	48	6.0	Li and F	Positive margins
Callery *et al* (1985)	25/33 years	54	4.0	9F, 5M, 1HPC, 2Le, 2D, 3Desmoid, 1Li, 2 SS	UK
Gutman *et al* (1994)	60/51 years	48	6.5	17A, 16SS, 10F, 6M, 3O, 2Li, 2Le, 1R, 3U	Size, multifocal lesions, vascular, lymphatic, skin or chest wall invasion
Johnstone *et al* (1993)	10/12 years	28	UK	4A, 2M, 1R, 1Li, 1SS, 1Sc	UK
Norris and Taylor (1968)	32/UK	49	4.0	5 OGS, 1 Le/R, 3 Li, 1D, 22F	Size, contour, atypia, mitotic activity
North *et al* (1998)	25/31 years	55	6.0	10A, 5SS, 3F, 2Li, 2Le, 1M, 1OGS, 1 U	Type of surgery
Oberman (1965)	13/30 y	56	7.1	7f, 3R, 2D, 1MM	Size, type of surgery
Pollard *et al* (1990)	25/81 years	55.4	5.9	11M, 6Li, 4F, 1CC, 1NS, 1Le, 1ASP	Type of surgery
Smola *et al* (1993)	8/23 years	56	12.8	2CHS, 1M, 2Li, 2F, 1A	UK
Stanley *et al* (1988)	4/UK	61	UK	2M, 2A	UK
Zelek *et al* (2003)	83/37 years	47	6.5	58M, 8A, 7L, 2Sc, 2R, 2OGS, 2Le, 2O	Grade, size

A = angiosarcoma; SS = stromal sarcoma; F = fibrosarcoma; M = malignant fibrous histiocytoma; Li = liposarcoma; D = dermatofibrosarcoma protuberans; Sc = spindle cell sarcoma; Cs = carcinosarcoma; Le = leiomyosarcoma; R = rhabdomyosarcoma; U = unspecified; CC = clear cell sarcoma; ASP = alveolar soft part sarcoma; MM = malignant mesenchymoma; OGS = osteosarcoma; Others = O.

) (Berg *et al*, 1962; Oberman, 1965; Norris and Taylor, 1968; Barnes and Pietruszka, 1977; Callery *et al*, 1985; Stanley *et al*, 1988; Pollard *et al*, 1990; Johnstone *et al*, 1993; Smola *et al*, 1993; Gutman *et al*, 1994; North *et al*, 1998; Barrow *et al*, 1999).

## MATERIALS AND METHODS

All cases diagnosed pathologically at our institution from 1910 to 2000 as breast sarcomas and stromal sarcomas were retrieved from Mayo Clinic Surgical Pathology files.

The H&E-stained sections were examined in all cases to confirm the diagnosis. An average of seven (range, 1–28) H&E slides per case were available. Clinical charts and surgical notes were retrospectively reviewed and the following information was collected: age, gender, size of tumour, clinical presentation, duration of symptom, history of radiation, type of surgery, local recurrences and systemic metastases. Follow-up information was obtained from patient records and death certificates. Patients with other prior primary malignancy in the breast, radiation therapy and metastatic disease to the breast were excluded.

Patients with cystosarcoma phyllodes were excluded, as well as patients with metaplastic carcinoma. For this purpose, immunoperoxidase studies were performed using two primary antibodies, vimentin, to determine immunocompetence and wide spectrum screening keratin, to diagnose a metaplastic carcinoma as reported earlier (Adem *et al*, 2002). In regards of the fact that some cases were diagnosed at the beginning of the century, if immunoperoxidase study with vimentin was negative, another block representative of the tumour was chosen for further immunostaining. If vimentin staining was still negative, search for an internal control such as normal or carcinomatous component was done in each case.

Size, diagnosis, infiltrative or nodular pattern, presence of heterologous elements, grade according to Broders' scheme of grading used at the Mayo Clinic, mitotic index (in 10 high-power fields, using a Leitz microscope, field diameter 0.45 mm), and necrosis were assessed.

Overall survival (OS) and cause-specific survival (CSS) following diagnosis were estimated based on the Kaplan–Meier method, overall and separately for morphological features. Associations between morphologic features and survival were evaluated univariately based on fitting Cox proportional hazards models. All calculated *P*-values were two-sided and *P*-values less than 0.05 were considered statistically significant.

## RESULTS

In all, 42 patients were retrieved between 1910 and 2000. Six were excluded after morphological review for the following reasons: cystosarcoma phyllodes (*n*=4), fibromatosis (*n*=1), benign haemangioma (*n*=1). Totally, 11 cases were also excluded after showing a positive stain with wide spectrum screening keratin, and being considered metaplastic carcinoma.

### Clinical data

Overall, 25 remaining patients constituted the study group and are summarised in Table 2

**Table 2:** Patients clinical and pathological characteristics in our series

	**Age (years)**	**Diagnosis**	**Duration**	**Surgery**	**Adjuvant therapy**	**Size**	**Gross**	**Margins**	**Grade**	**Local recurrence**	**Metastases**	**Last follow-up**
Case 1	38	MXFS	15 m	R Mast	N	UK	C	N	2	3 y, S	N	DUK, 45 m
Case 2	38	F	UK	R Mast	RT	5	UK	UK	2	N	N	DOC, 5.5 y
Case 3	31	PS	2 m	Excision	N	UK	UK	I	4	1 y, R Mast	N	DOC, 1 y
Case 4	38	A	UK	Excision	N	UK	UK	I	2	5 times, 3 to 6 y, S/RT	L, 6 y	DOD, 84 m
Case 5	72	PS	UK	R Mast	N	3	I	I	4	N	N	Alive, 18 y
Case 6	49	F	1 m	Mast	UK	3	UK	I	3	N	N	DOC, 37 y
Case 7	43	A	1 m	Mast	N	8	C	I	1	N	B/L, 1 y, S/CT	DOD, 16 m
Case 8	48	MXFS	96 m	Mast	RT	5.5	I	I	3	4 m, No TTT	N	AWD, 6 m
Case 9	55	F	2 m	Mast	UK	UK	UK	N	2	Twice, 11 m and 17 m, S	N	DOD, 76 m
Case 10	67	Le	UK	Excision	CT	2	UK	UK	4	N	Li/B/Skin, at presentation, CT	DOD, 7 m
Case 11	39	A	2 m	Excision	N	8	I	I	2	20 m, S	Li/Jejunum, 9 y, None	DOD, 114 m
Case 12	32	PS	2 m	Excision	N	UK	UK	I	4	2 m, S	N	DOD, 23 m
Case 13	52	F	4 m	M R Mast	N	4.5	C	I	3	N	N	Alive, NED, 23.5 y
Case 14	27	A	11 m	S Mast	N	12	I	I	2	11 m, UK	L/Li/S, 22 m, UK	DOD, 32 m
Case 15	63	MXFS	30 y	S Mast	N	4	C	N	2	N	N	DOC, 21 y
Case 16	60	PS	12 m	R Mast	RT	10	C	N	4	N	B/Lu, 6 y, RT	DOD, 88 m
Case 17	55	Le	UK	Mast	N	4	UK	N	4	N	Multiple sites, 6 y, CT	DOD, 77 m
Case 18	33	A	12 m	R Mast	RT	10	C	I	2	10 m, UK	B, 10 m, RT	DOD, 13 m
Case 19	33	HPC	UK	UK	UK	UK	UK	N	4	N	L/Li/Pelvis, UK, RT	DOD, 41 m
Case 20	24	PS	12	S Mast	N	5	C	I	3	N	N	Alive, NED, 11 y
Case 21	32	A	11 m	S Mast	N	UK	UK	I	3	14 m, RT	B/L, 14 m, RT/CT	DOD, 26 m
Case 22	42	F	UK	S Mast	N	3	UK	I	3	8 m, S	N	DOC, 49 y
Case 23	54	F	1 m	M R Mast	N	5	UK	N	2	N	N	Alive, NED, 13 y
Case 24	81	PS	UK	M R Mast	N	0.3	UK	I	4	N	N	Alive, NED, 14 m
Case 25	54	OGS	UK	M R Mast	N	10	C	I	3	N	N	Alive, NED, 4 y

*Abbreviations*: MXFS = myxofibrosarcoma; F = fibrosarcoma; PS = pleomorphic sarcoma; AGS = angiosarcoma; Le = leiomyosarcoma; HPC = hemangiopericytoma; OGS = osteosarcoma; UK = unknown; R = radical; Mast = mastectomy; S = simple; M = modified; RT = radiotherapy; CT = chemotherapy; C = circumscribed; I = infiltrative; y = year; m = month; DOC = dead of other causes; DUK = dead of unknown cause; NED = no evidence of disease; DOD = dead of disease; Lu = lung; B = bone; Li = liver; S = spleen; N = nodular or pushing margins; I = infiltrative.

. There were 25 women age range 24–81 (mean 45 years). In total, 24 cases presented with lump, two of them associated with pain. In one case, it presented as an incidental mammographic finding. Contralateral breast sarcoma had been diagnosed elsewhere 3 years earlier in one case, renal cell carcinoma 5 years later in one case, colon cancer 4 years earlier in one case, skin melanoma and uterine cancer in one case 16 and 27 years earlier, respectively. No history of prior radiation was found in any case, therefore excluding postradiation sarcoma. The duration of symptoms for 16 patients ranged between 1 month to 40 years (mean 3.2 years).

Surgical treatment was excision in five cases, mastectomy in 19 cases (modified, four; simple, five; radical, five; not specified, five), and unknown in one case. Adjuvant therapy was administered in five cases (radiation, four; chemotherapy, one).

The right breast was affected in 10 cases, while the left was affected in 15 cases.

### Pathological data

Gross description was available in 12 cases. Eight tumours were described as well-circumscribed, four as infiltrative of which two were angiosarcoma. Tumour size was available on 18 patients, and the mean tumour size was 5.7 cm (range 0.3–12.0). Angiosarcomas tended to be larger in size with a mean of 10 cm (range, 8–12 cm).

After present review, histopathological diagnoses were fibrosarcoma (*n*=6), angiosarcoma (*n*=6), pleomorphic sarcoma (*n*=6), leiomyosarcoma (*n*=2), myxofibrosarcoma (*n*=3), hemangiopericytoma (*n*=1) and osteosarcoma (*n*=1). Tumours were graded as low grade (grade 1, one; grade 2, nine), and high grade (grade 3, seven; grade 4, eight). Necrosis was observed in four cases (three high-grade tumours). In all, 11 (range, 0–43) mitoses were found on average in 10 HPF. Heterologous component was seen in one case of osteosarcoma. Seven had pushing margins while 16 had infiltrative ones.

An *in situ* ductal carcinoma component was observed in one case. In this case of pleomorphic sarcoma, keratin staining was negative in neoplastic cells with adequate internal control (the *in situ* component as well as benign entrapped ducts).

There was no metastasis in the 15 cases where axillary node dissection was performed.

### Follow-up and survival analysis (Figure 1)

**Figure 1:**
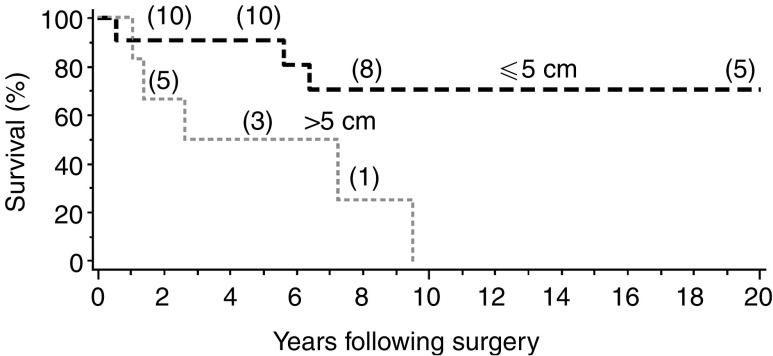
Overall survival following surgery, according to tumour size (⩽5 *vs* >5 cm). The numbers in parentheses indicate the number of patients still at risk at selected time points.

Overall mean and median follow-up were, respectively, 10.5 and 6.4 years (range, 7 months–41 years). Local recurrence was observed in 11 patients and ranged from 2 to 36 months (mean 15 months), while distant metastasis was observed in 10 patients, in order of frequency affecting the lungs (*n*=7), bones (*n*=6), liver (*n*=5), spleen (two) and skin (two). In one case, other sites were also kidney, pancreas, adrenal, omentum, epicardium and mediastinum. Of the 25 patients, 12 have died of disease and six of other causes. At the last follow-up, seven patients were still alive with a mean and median follow-up of 10.2 and 10.9 years, respectively.

Five-year overall (OS) and cause-specific survival (CSS) were 66 and 70%, respectively. Five-year OS and CSS were both 91% for tumours ⩽5 cm, and 50% for tumours >5 cm. Tumour size was significantly associated with OS (risk ratio=1.3 per 1 cm increase; 95% CI, 1.02–1.7; *P*=0.036). There was no significant difference between low- and high-grade lesions (OS were 60 and 70%, *P*=0.14, CSS were 70 and 70%, *P*=0.5, respectively) or tumours showing infiltrative compared to pushing margins (OS were 65 and 71%, *P*=0.47, CSS were 65 and 86%, *P*=0.94, respectively) in terms of OS or CSS.

Although there was no statistically significant association between tumour size and metastasis or recurrence, mean tumour size of patients with recurrence or metastasis was 7.7 cm, compared to 4.9 and 4.3 cm, respectively, for patients without recurrence or metastasis. Four out of five patients treated with simple excision had recurrence or metastasis.

By the most common histopathologic types, all but one patient with angiosarcoma (4/5), one patient with fibrosarcoma, and two patients with pleomorphic sarcoma, died of disease.

## DISCUSSION

Primary breast sarcomas are extremely rare (Moore and Kinne, 1996). In our institution, they compose 0.0006% of breast malignancies. They constitute a specific clinicopathologic entity and, therefore should be differentiated from the two main entities in differential diagnosis, cystosarcoma phyllodes and metaplastic carcinoma. Specific morphological features (biphasic tumour, with leaf-like architecture and epithelial component) recognise the former, and extensive sampling of the tumour can help when a stromal overgrowth is present. The latter is recognised on H&E sections by the presence of a carcinomatous component, or based on a cytokeratin immunopositivity of the neoplastic spindle cells.

Reported series in the English literature had included all three entities as breast sarcomas, and include in their reports angiosarcomas, desmoid tumours, and lymphosarcomas (Botham *et al*, 1958; Berg *et al*, 1962; Oberman, 1965; Donegan, 1967; Fawcett, 1967; Kennedy and Biggart, 1967; Norris and Taylor, 1968; Rissanen and Holsti, 1968; Gogas *et al*, 1976; Barnes and Pietruszka, 1977; Ludgate *et al*, 1977; Khanna *et al*, 1981; Callery *et al*, 1985; Christensen *et al*, 1988; Stanley *et al*, 1988; Terrier *et al*, 1989; Pollard *et al*, 1990; Pitts *et al*, 1991; Ciatto *et al*, 1992; Luna Vega *et al*, 1992; Johnstone *et al*, 1993; Smola *et al*, 1993; Gutman *et al*, 1994; McGregor *et al*, 1994; Moore and Kinne, 1996; North *et al*, 1998; Barrow *et al*, 1999; McGowan *et al*, 2000). Therefore, reliable assessments of prognostic factors are difficult to make based on the published literature. Table 1 depicts comparable major series using soft-tissue tumours as basis for classification.

Tumour size seems to be the most frequently reliable prognostic factor in many of these series, as in breast carcinomas and soft-tissue sarcomas (Oberman, 1965; Norris and Taylor, 1968; Gutman *et al*, 1994; Barrow *et al*, 1999; Zelek *et al*, 2003) Other reported prognostic factors are the histopathological diagnosis (Barrow *et al*, 1999), the infiltrative features (Norris and Taylor, 1968; Barnes and Pietruszka, 1977), the histopathologic grading (Norris and Taylor, 1968; Barnes and Pietruszka, 1977; Gutman *et al*, 1994; Barrow *et al*, 1999; Zelek *et al*, 2003), presence of positive margins (Berg *et al*, 1962; Barrow *et al*, 1999), and extent of surgery for local recurrence (Pollard *et al*, 1990; North *et al*, 1998). Some authors found age to be of prognostic importance (Ludgate *et al*, 1977). Margins status is a major risk factor for recurrence as it occurs in any neoplastic entity, and some authors advised adjuvant radiotherapy for cases with positive margins (Callery *et al*, 1985; Smola *et al*, 1993), or less than 2 cm of clear margins (McGowan *et al*, 2000). Treatment is generally based on a wide local excision, without axillary dissection (Barrow *et al*, 1999). Breast sarcomas bear different histogenesis than breast carcinomas as shown by cytogenetic studies (Garcia-Palazzo *et al*, 1992), and biological behaviour (Berg *et al*, 1962).

We believe that breast sarcomas are comparable to soft-tissue sarcomas seen elsewhere. They present mainly as a lump and size is a prognostic marker with 5 cm serving as a valuable cut point. Tumour grade did not correlate with the outcome in our series but statistical power was limited and this finding could be related to the small size of the series. Lymphatic spread is uncommon as shown by the absence of axillary lymph node metastasis in our cases, and therefore axillary node dissection is not necessary. When lymph node metastasis is present, the diagnosis of a metaplastic carcinoma should be considered even in the presence of a pure spindle cell neoplasm.
